# The Behavior Rating Inventory of Executive Function-Preschool Version in Nepali Children With Poor Growth in Infancy

**DOI:** 10.1177/30502225251338633

**Published:** 2025-05-26

**Authors:** Roshan Sintakala, Suman Ranjitkar, Ram K. Chandyo, Ingrid Kvestad, Manjeswori Ulak, Jaya S. Silpakar, Merina Shrestha, Laxman Shrestha, Mari Hysing

**Affiliations:** 1 Department of Pediatrics, Child Health Research Project, Institute of Medicine, Tribhuvan University, Kathmandu, Nepal; 2 Department of Community Medicine, Kathmandu Medical College, Nepal; 3 Regional Centre for Child and Youth Mental Health and Child Welfare, NORCE Norwegian Research Centre, Bergen, Norway; 4 Innlandet Hospital Trust, Lillehammer, Norway; 5 Center for Intervention Science in Maternal and Child Health, Department of Global health and primary care, Centre for International Health, University of Bergen, Bergen, Norway; 6 Department of Psychosocial Science University of Bergen, Norway

**Keywords:** executive functions, BRIEF-P, preschool, confirmatory factor analysis, psychometrics

## Abstract

**Objectives:**

To describe psychometric properties of the Behavior Rating Inventory of Executive Function-Preschool (BRIEF-P) and its discriminatory ability for risk groups for executive dysfunction (low birth weight (LBW), stunting and low maternal education) among children with poor growth in infancy.

**Introduction:**

BRIEF-P is widely used to measure executive functions, but knowledge on its psychometric properties in low-and-middle-income countries is limited.

**Method:**

The sample consisted of 529 parents of children (30-35 months). BRIEF-P was subject to confirmatory factor analysis, and internal consistency by alpha calculations and inter-correlations.

**Results:**

A good fit for a bifactor-model with 5 subscales and a general factor. BRIEF-P subscales were higher when mothers had low versus high education. Children born with LBW compared to normal birth weight had poorer Shift and Flexibility scores.

**Conclusion:**

BRIEF-P is feasible and reliable tool to measure executive functions in Nepali children and is associated with key risk factors for executive dysfunction.

## Introduction

Executive functions (EFs) are a complex set of mental processes that control and monitor cognitive, emotional and behavioral functions.^1^ EFs are a set of higher-order mental processes such as cognitive shifting (the ability to flexibly switch the focus of attention based on the demands of the situation),^2^ planning (the ability to develop a sequence of steps needed to carry out a task and reach a goal),^3^ working memory (the ability to store and manipulate information temporarily),^4^ inhibitory control (the ability to override a pre-potent or dominant response),^5^ and emotional control (the regulation of one’s emotional state and feelings according to the demand of the situation).^6^ Hence, EFs are essential for achieving goals within the complex changing environment and are required for novel tasks, paying attention, and shifting from one task to another.^7^ During the preschool years, there is a rapid development of executive functions,^8^ and thus this is an important time period for the assessment of EFs which can assist in determining if children need support to improve their EFs.^9,10^ Additionally, executive dysfunction must be detected early to prevent negative effects on school performance, social function, self-esteem, and quality of life.^11^

Preschool children in low–and-middle-income countries (LMICs) are likely to have more of the established risk factors for EFs.^12-15^ The present study is performed in Bhaktapur, Nepal. Approximately 40% of Nepali children live in poverty with poor health and low standard of living.^16^ Moreover, children in Nepal experience higher rates of a range of risk factors for executive dysfunction such as stunting, premature birth and low birth weight.^17,18^

Preterm birth and low birth weight are known risk factors for executive dysfunction.^19,20^ For instance, pre-term born children have shown to have higher scores in the emotional control and working memory subscales.^20^ In a study of preschool children, increments in birth weight, even among children born with normal weight, was associated with improved executive functioning.^21^ Further, elevated EF scores on the BRIEF-P have been found among children characterized by malnutrition and stunting in a study from India.^22^ A social gradient in EF has also been identified, in which maternal education is positively correlated with EFs, mainly in Planning and Working Memory.^23^

While children from LMICs are at greater risk of executive dysfunction,^24,25^ most instruments used to assess executive function have been developed in Western population.^26^ Therefore, less is known of the nature, quality and properties of tools developed for the LMICs samples.^27^ In addition, there is no adequate adaptation and a lack of evidence on cross-cultural validity and the quality of psychometric properties for tools that measure EFs in LMIC settings.^28^

The Behavior Rating Inventory of Executive Function-Preschool version (BRIEF-P)^29^ is a widely used instrument to measure EFs from 2 years through 5 years. The instrument includes 5 subscales (Inhibit, Shift, Emotional Control, Working Memory, and Planning and Organization) constituting the Global Executive Composite scores (GEC) and 3 indices (Inhibitory Self-Control Index (ISCI), Flexibility Index (FI), and the Emergent Metacognition Index (EMI). It has been discussed whether EFs are best viewed as a unitary or a multidimensional construct,^30^ and this is also reflected in the studies that have looked into the factor structure of the BRIEF-P. The original 5 factor structure, after excluding 4 items, has shown adequate fit by confirmatory factor analysis along with satisfactory internal consistency and moderate validity.^31^ A Catalan version was not able to measure the goodness of fit for either a 5 factor model or a unidimensional model from both parents’ and teachers’ data.^32^ The tool has shown good internal consistency and convergent ability among typically developed preschoolers.^33^ A 3 factor structure was also supported in children with neurodevelopmental disorders.^34^ BRIEF-P scales has shown associations with corresponding EF measures which supports it validity.^35^ This study involves children from the low- and middle-income setting of Bhaktapur municipality, Nepal. Most families in Bhaktapur are traditionally engaged in agriculture and the community is relatively traditional, cultural capital, and heritage city.^36^ Children in the community are exposed to multiple risk factors such as malnutrition, stunting, infectious disease that may affect their development of EFs.^25^

The aim of the current study is to assess the psychometric properties of a translated version of the BRIEF-P in Bhaktapur, Nepal. Additionally, to identify the discriminatory abilities of BRIEF-P for children groups that have increased risk of executive dysfunction (a) are stunted, (b) are born with low birth weight, and (c) with mothers having low educational levels compared to reference groups.

## Method

### Participants

The participants were parents of 529 children (92% of the total available cohort of 600), 30 to 35 months old who participated in the follow-up of a randomized, double-blind, placebo-controlled trial.^37^

For the main study, we conducted a door-to-door survey to identify households with children aged 6 to 11 months and physicians and field supervisors screened the children for eligibility. In the main study, we screened a total of 733 participants, and excluded a total of 133 children according to the following exclusion criteria: Plan to migrate within a year (n = 7), Acute/chronic illness (n = 41), Taking multivitamins (n = 19), No consent (n = 18), Length for age z score >−1 z score (n = 41), and Weight for length <−3 z score (n = 7). Children with a length- for-age less than −1 z-score who resided in Bhaktapur municipality and surrounding communities and with informed consent from the caregiver, were included in the main study. Children with severe illness, those who were taking vitamin supplements that included vitamin B12 and children with severe anemia were excluded. At the end of the 1 year of supplementation, 574 were available for follow-ups (dropout n = 26). Among 574 children, with 45 additional drops out due to migration and refusal, 529 participated in the current study. And shown that children’s development and growth were not affected by vitamin B12 supplementation in a main study.^38,39^

### Procedure

We followed the STROBE Checklist for our research (Supplementary material). The data was gathered from January 2017 to December 2019. Demographic information was collected by fieldworkers when the children were 6 to 11 months, including parental education and whether family stayed in a joint family, resided in a rented house, owned land and received remittance from abroad. Maternal education was categorized into less or more than 10 years of education which is the secondary level and above.

Birth weight was collected through parental report based on hospital delivery records. Low birth weight was defined as a birth weight less than 2500 g. Length was measured using infantometers and weight from an electronic scale (Salter/HoMedics Group, UK and seca, Germany) by field supervisors. Length was converted to z-scores according to the WHO growth chart^40^ and stunting was defined as length-for-age z-scores below −2.

### Instrument

Parental reported BRIEF-P was administered by trained psychologists in distraction-free assessment rooms. BRIEF-P contains 63 statements, where parents responds to how often a specific behavior has been a problem during the past 6 months by rating “Never” (1), “Sometimes” (2), or “Often” (3).^29^ BRIEF-P has 5 subscales: Inhibit (16 items), Shift (10 items), Emotional Control (10 items), Working Memory (17 items), and Plan and Organize (10 items). These 5 subscales represent the Global Executive Composite scores (GEC) and are further divided into 3 broad indices: Inhibitory Self-Control Index (ISCI; Inhibit and Emotional Control), Flexibility Index (FI; Shift and Emotional Control), and Emergent Metacognition Index (EMI; Working Memory and Plan and Organize). Higher scores on the scales indicate more executive dysfunction. The raw scores were converted into standardized T-scores based on American norms with a mean (SD) of 50 (10). The American norms are from a representative sample that covers a range of ethnicities, socio-economic classes, and population densities. The normative sample has 2 age groups: 2 to 3 years and 4 to 5 years and the norms are gender specific.^41^

### Translation and Cultural Adaption

The English version of the BRIEF-P was translated into Nepali language by a psychologist and medical doctors and then back translated by a qualified person not involved in the study and fluent in both Nepali and English language, following standard guidelines for translation processes. Discrepancies were discussed and adjusted. For some words, we chose the nearest similar words in Nepali due to the lack of exact vocabulary in the Nepali language. For example, for the item “Is impulsive” we could not find an exact Nepali translation that gave an acceptable back translation. We therefore translated to a similar word in Nepali in accordance with the developers (Psychological Assessment Resources (PAR)) who also approved the final translated version.

### Statistical Analysis

We present descriptive statistics as numbers, percentages, means, and standard deviations (SD). Raw and composite scores for each subscale and index scores are presented by means (SD) along with confidence intervals (CI) for boys and girls separately. To compare the raw and composite subscale and index scores of the study sample with the American norms, we used student *t*-tests. Internal consistency of the test was measured by Cronbach’s alpha for all subscales and through inter-correlations of the subscale raw scores by Pearson’s correlation coefficients. Alpha values <.6, .6 to .8, and >.8 were set as questionable, acceptable, and good, and the value of effect size was evaluated as very small (0.01), Small (0.20), medium (0.50), Large (0.80), very large (1.20), and huge (2.0).

The 63 BRIEF-P items were submitted to confirmatory factor analysis (CFA) with the JASP statistical analytical program using weighted least squares mean, and variance (WLSMV) adjusted for the categorical data method of estimation due to the highly skewed data (ordinal with 3 options).

The model that was considered was a 5-first-order-factor model. The 5 first-order factors were the 5 subscales Inhibition, Emotional Control, Shift, Working Memory, and Plan and Organize. Then a bi-factor model was assessed including a general factor in addition to the 5 factors.

Model fit was assessed by comparative fit index (CFI), and root mean square error of approximation (RMSEA). In the current study, CFI values greater than 0.90,^42^ together with RMSEA values less than 0.08 were considered acceptable, although CFI values above 0.95 and RMSEA below 0.06 were preferred.^43^ The statistical analyses were performed in STATA (version 16) and the confirmatory factor analysis was carried out in JASP (Version 0.14.0).

### Ethics

Ethical clearances were obtained from the Nepal Health Research Council (NHRC; No. 73/2017) in Nepal and from the Regional Committee for Medical and Health Research Ethics (REC; No.2014/1528) in Norway. All participants gave written informed consent.

## Results

The demographic characteristics of the studied sample are reported in Table 1.

**Table 1.** Socio-Demographic and Clinical Characteristics of the Sample of Mildly Stunted **Nepali** Children (n = 529).

Characteristics	Number	%	Mean	SD
*Socio-demographic status:*				
*Family staying in joint family*	253	47.8		
*Family residing in rented house*	252	47.6		
Family having own land	249	47		
Remittance from abroad	52	9.8		
Mother’s education				
Up to grade10	295	55.7		
Above grade10	234	44.3		
*Child characteristics:*				
Age of child (months)			31.9	2.2
*Sex of the child (Male)*	275	52		
*Age of play group admission (month;* n = 474)			20.5	5.1
*Age of school nursery admission (month;* n = 275)			27.9	3.9
Admission at government school	145	27.4		
*Perinatal characteristics:*				
Birthweight, (in grams)			2787	497
*Preterm birth (<37 weeks of gestation)*	52	9.8		
Low birth weight (<2500 gm)	108	20.4		
*Stunting (length for age Z score <−2) at 6 to 11 months*	179	33.8		

The mean (SD) age of the children was 31.9 (2.2) months and 52% were male. Approximately 10% were born preterm and 20% had a birth weight less than 2500 g. One third of the infants were stunted at baseline when they were 6 to 11 months of age. Out of the 529 respondents, 482 mothers,31 fathers and 16 other caregivers completed the BRIEF-P questionnaire.

### BRIEF-P Scores and Comparison to American Norms

The BRIEF-P mean (SD) raw scores and T-scores according to American norms for the subscales and indices are shown in Table 2. Mean T-scores were above the mean of 50 for all subscales and indexes for both boys and girls, ranging from 51.1 to 63.8 for boys and 54.5 to 67.4 for girls. Our results show that T-scores are significantly higher than American norms for both boys and girls except for the Plan and Organize subscales in the boys.

**Table 2.** Participant Raw Scores and T-scores of the BRIEF-P Subscales and Indexes in 529 Nepali Children.

BRIEF-P	Boys: n = 275				Girls: n = 254			
	Raw Score	T score		*P*-value^*^	Raw Score	T score		*P*-value^*^
	Mean (SD)	Mean (SD)	95% CI		Mean (SD)	Mean (SD)	95% CI	
Inhibition	30.9 (6.1)	60.5 (10.9)	59.2-61.9	<.001	30.2 (6)	66.6 (12.6)	65.1-68.1	<.001
Shift	16.8 (4.1)	54.4(10.1)	53.2-55.7	<.001	17.5 (3.9)	60.1 (11.9)	58.6-61.7	<.001
Emotional control	16.8 (3.7)	52.4(8.6)	51.4-53.5	<.001	17.1 (3.7)	54.5(9.1)	53.4-55.6	<.001
Working memory	30.9 (5.8)	63.8(11.6)	62.3-65.1	<.001	30.8 (5.5)	67.4(11.6)	65.9-68.8	<.001
Plan and organize	16.2 (3.7)	51.1(11.5)	49.8-52.5	.068	16.1 (3.2)	54.8(10.3)	53.5-56.1	<.001
EMI^a^	47.1 (8.8)	59.2(11.5)	57.9-60.7	<.001	46.8 (7.9)	63.5 (11.1)	62.2-64.9	<.001
ISCI^b^	47.8 (8.8)	57.8(9.9)	56.6-58.9	<.001	47.4 (8.8)	62.2 (11.2)	60.8-63.6	<.001
FI^c^	33.6 (7.1)	53.8(9.8)	52.7-54.9	<.001	34.7 (6.8)	58.2 (10.7)	56.9-59.5	<.001
GEC^d^	111.6 (19.4)	59.1(11.3)	57.8-60.5	<.001	111.7 (18.3)	64.7(11.8)	63.2-66.1	<.001

a Emergent Metacognition Index.

b Inhibitory Self-Control Index.

c Flexibility Index.

d Global Executive Composite score.

* Compared with the means of American norm.

### Factor Structure

The fit of the first-order model was good with CFI = 0.961 and RMSEA = 0.031. A bi-factor solution was tested with the 5 first-order factors and a general factor, showing a good fit with a CFI = 0.970 and RMSEA = 0.029. The factor loadings were higher on the general factor than on the specific subscales.

### Reliability

The Cronbach’s alpha values indicate acceptable to good internal consistency for all 5 subscales and the 4 indices, ranging from .68 to .92. The subscale inter-correlations were all positive and significant, ranging from 0.44 to 0.70 (Table 3).

**Table 3.** Internal Consistency of the BRIEF-P Subscales and Indexes in **Nepali** Children.

Subscales and indexes											
		α^a^	Intercorrelations^b^								
Subscales			1	2	3	4	5	6	7	8	9
*1*	*Inhibit*	0.78	1								
*2*	*Shift*	0.71	0.47*	1							
*3*	*Working memory*	0.76	0.58*	0.61*	1						
*4*	*Emotional control*	0.68	0.70*	0.59*	0.56*	1					
*5*	*Plan and organize*	0.68	0.60*	0.47*	0.44*	0.66*	1				
*Indexes*											
*6*	*Inhibitory self-control*	0.81	0.93*	0.58*	0.82*	0.72*	0.60*	1			
*7*	*Flexibility*	0.81	0.58*	0.90*	0.89*	0.64*	0.51*	0.78*	1		
*8*	*Emergent metacognition*	0.85	0.72*	0.60*	0.56*	0.95*	0.86*	0.74*	0.65*	1	
*9*	*GEC* ^c^	0.92	0.86*	0.75*	0.76*	0.88*	0.76*	0.92*	0.84*	0.91*	1

a Chronbach’s Alpha >.8, Good; 0.7 < 0.8, Acceptable; 0.6 < 0.7, Questionable.

b Pearson’s correlation coefficient.

c Global Executive Composite score.

* significant at *P* values <.05.

### Executive Function in High-Risk Groups

Children born with low birth weight had poorer scores on the Shift subscale (*P* = .01) and Flexibility index (*P* = .04). Children of mothers with low education (<10 grades of schooling) had higher scores on all subscales compared with the reference group. There were no differences in the BRIEF-P scores according to whether the children were stunted or not during infancy (Table 4).

**Table 4.** BRIEF-P Raw Scores According to High-Risk Groups in Nepali Children.

Risk Groups	N	Inhibit				Shift				Emotional control			
		Mean diff.	Mean (SD)	*P*-value	d^a^	Mean diff.	Mean (SD)	*P*-value	d^a^	Mean diff.	Mean (SD)	*P*-value	d^a^
*Birthweight*													
*<2500 g*	108	0.22	30.78(5.99)	.739	0.03	1.13	18.05(4.26)	.008	0.27	0.39	17.31(3.89)	.3	0.
*≥2500 g*	421		30.57(6.12)				16.92(3.92)				16.92(3.65)	19	10
*Stunted* ^f^													
*Yes*	179	0.53	30.94(6.08)	.346	0.08	0.06	17.19(4.12)	.867	0.01	0.16	17.10(3.63)	.6	0.
*No*	350		30.42(6.09)				17.13(3.97)				16.93(3.75)	37	19
*Maternal education*													
*Up to grade 10*	295	1.50	31.28(5.9)	.004	0.25	1.04	17.61(4.04)	.003	0.26	1.01	17.4(3.5)	.001	0.27
*Above grade 10*	234		29.76(6.2)				16.57(3.91)				16.4(3.9)		
Risk Groups	N	Working memory				Plan/Organize				EMI^b^			
		Mean diff.	Mean (SD)	*P*-value	d^a^	Mean diff.	Mean (SD)	*P*-value	d^a^	Mean diff.	Mean (SD)	*P*-value	d^a^
*Birthweight*													
*<2500 g*	108	0.24	30.99(5.59)	.689	0.04	0.04	16.11(3.36)	.917	0.01	0.21	47.10(8. 04)	.819	0.
*≥2500 g*	421		30.75(5.70)				16.15(3.48)				46.89(8. 47)		02
*Stunted* ^f^													
*Yes*	179	0.28	30.97(6.05)	.591	0.05	0.48	16.46(3.57)	.134	0.09	0.76	47.43(8. 94)	.326	0.
*No*	350		30.69(5.49)				15.98(3.39)				46.67(8. 09)		09
*Maternal education*													
*Up to grade 10*	295	1.15	31.3(5.4)	.020	0.21	0.80	16.5(3.5)	.007	0.27	1.90	47.8(8.20)	.007	0.29
*Above grade 10*	234		30.1(5.9)				15.6(3.27)				45.8(8.4)		
Risk Groups	N	ISCI^c^				FI^d^				GEC^e^			
		Mean diff.	Mean (SD)	*P*-value	d^a^	Mean diff.	Mean (SD)	*P*-value	d^a^	Mean diff.	Mean (SD)	*P*-value	d^a^
*Birth weight*													
*<2500 g*	108	0.62	48.10(8.93)	.515	0.07	1.53	35.37(7.36)	.041	0.22	1.96	113.26(18.39)	.335	0.10
*≥2500 g*	421		47.48(8.75)				33.83(6.80)				111.30(18.94)		
*Stunted* ^f^													
*Yes*	179	0.69	48.04(8.74)	.395	0.08	0.22	34.29(6.93)	.727	0.03	1.51	112.67(19.36)	.38	0.
*No*	350		47.36(8.82)				34.07(6.96)				111.16(18.59)	4	08
*Maternal education*													
*Up to grade 10*	295	2.50	48.7(8.4)	.001	0.31	2.10	35.1(6.7)	.007	0.24	5.50	114.1(18.4)	.007	0.29
*Above grade 10*	234		46.2(9.1)				33.0(6.9)				108.6(18. 9)		

a Cohen’s D.

b Emergent Metacognition Index.

c Inhibitory Self-Control Index.

d Flexibility Index.

e Global Executive Composite score.

f 6t o 11 months of age length for age <−2 z score.

## Discussion

In the present study, we measured the psychometric properties of a Nepali version of BRIEF-P among 529 preschool children with poor growth in infancy. Confirmatory factor analysis demonstrated a good fit for both a first order factor and a bi-factor model. The results showed poorer EF in children with mothers with low education, and partly for low birth weight depicting the ability of the Nepali version of BRIEF-P to distinguish low- and high-risk groups. However, there were no differences between stunted children and not stunted during infancy.

The internal consistency of the BRIEF-P subscales was acceptable to good with alpha values very close to those previously reported, both in the normative sample^44^ and in other studies that included children aged 2 to 6 years.^32^ The factor model confirmed in the present study is in close alignment with a first order 5 factor model and a second order factor in a Spanish community-based study of the BRIEF-P.^31^ Our findings are also in line with a study of BRIEF in school age children providing support for a bi-factor model with the 5 first-order factors and a general factor.^45^

The mean subscale and index standardized scores were all in the range of average (T score of 50) to close to 2 SDs above the mean, indicating that poor EF is common in the present sample compared to US norms. However, caution is recommended while comparing with US norms since the validity of the American norms for our study setting is uncertain.^22^ At the same time, previous findings in this sample have shown poor function on cognitive tests such as the Bayley Scales of Infant and Toddler Development,^46^ supporting the findings of impaired EF among these children.

The BRIEF-P showed the expected ability to discriminate between high-risk groups. In line with the current findings, parental socio-economic status with a composite of maternal and paternal education and occupation status using Hollingshead index^47^ was associated with EF in a twin study from the United States.^48^ Similarly, research by Hackman et al and his colleagues found lower maternal education predicted worse performance in planning and working memory subscales.^23^ One fifth of the children in the current sample was born with low birth weight. We found significantly weaker scores on the Shift and Flexibility Index scales in these children compared to those born with normal birth weight. A study from United States showed similar results where infants born with low birth weight were at higher risk of poor executive functioning than those born with normal birth weight.^21^

## Strengths and Limitations

An important strength of this study is the large sample size of parents of 529 children. The BRIEF-P questionnaire was translated and back translated according to standard translation procedures and carried out by trained medical doctors and skilled psychologists. Due to time restrictions, the translated version was not discussed with parents, which would have given valuable information. We have only included 1 measure of EF, and future studies should include a broader range of measures. The main limitation is that the sample is not representative of the total population since it includes mildly stunted children participating in a randomized clinical trial. As a consequence, our findings may not be generalizable to the Nepalese population as a whole. The present study was not specifically designed for psychometric evaluations, and thus no sample size calculation was done in this regard.

## Conclusion

The original factor structure of the BRIEF- P was confirmed in the present study in Nepali children. The instrument also discriminated between high-risk groups, such as those defined by maternal education and birthweight. While this is an early indication that the BRIEF-P can be used among high-risk children in the current Nepali setting, more studies are needed and preferably in population-based study samples to confirm our findings.

## Supplemental Material

sj-docx-1-gph-10.1177_30502225251338633 – Supplemental material for The Behavior Rating Inventory of Executive Function-Preschool Version in Nepali Children With Poor Growth in Infancy Supplemental material, sj-docx-1-gph-10.1177_30502225251338633 for The Behavior Rating Inventory of Executive Function-Preschool Version in Nepali Children With Poor Growth in Infancy by Roshan Sintakala, Suman Ranjitkar, Ram K. Chandyo, Ingrid Kvestad, Manjeswori Ulak, Jaya S. Silpakar, Merina Shrestha, Laxman Shrestha and Mari Hysing in Sage Open Pediatrics
